# Fabrication of nitrogen-doped nano-onions and their electrocatalytic activity toward the oxygen reduction reaction

**DOI:** 10.1038/s41598-017-04597-6

**Published:** 2017-06-23

**Authors:** E. Y. Choi, C. K. Kim

**Affiliations:** School of Chemical Engineering & Materials Science, Chung-Ang University, 221 Huksuk-dong, Dongjak-gu, Seoul, 156-756 Korea

## Abstract

Nitrogen-doped nano-onions (NNO) were prepared as electrocatalytic materials for the oxygen reduction reaction (ORR). The nano-onions (NO), spherical graphitic material particles, were prepared by pyrolysis of nanodiamonds (ND). Oxidized NO (ONO) was prepared from NO by a modified Hummers’ method, and this was mixed with urea, followed by pyrolysis, resulting in the formation of NNO. The nitrogen content and molar ratio of nitrogen-containing groups in the NNOs were varied by controlling the oxygen content of ONO to explore the effect of nitrogen content on the ORR activity. The formation of NO was confirmed by Raman spectroscopy, X-ray diffraction analysis, and high-resolution transmission electron microscopy. X-ray photoelectron spectroscopy analyses were conducted to confirm the formation of the NNO and the structures of the nitrogen-containing groups in the NNOs. The ORR activities of the NNOs were investigated using a rotating disk electrode. The NNOs showed a higher onset potential than that of NO, and the ORR activity of the NNO could be improved by increasing the number of active sites (nitrogen-containing groups) in the NNO. In addition, the NNO exhibited better long-term stability and resistance toward methanol crossover in the ORR than the platinum-based catalysts.

## Introduction

The oxygen reduction reaction (ORR) of a fuel cell is a pivotal performance-determining factor. Platinum-based catalysts are commonly used in fuel cells to achieve high electrocatalytic activity for the ORR^1, 2^. However, the high price and scarcity of Pt and the low stability of Pt-based catalysts limit their applications. Consequently, alternatives to Pt-based catalysts that are inexpensive, highly active, and stable are required. Advances in the development of electrocatalytic materials for ORR such as alloys^3–5^, nitrides^6–8^, foreign atom doped carbon-based materials^9, 10^, and their complexes with metal^11, 12^ have been reported recently.

As efficient metal-free catalysts, nitrogen doped carbon-based materials (NC) have attracted considerable attention in the research community^13–23^. Nitrogen doping of carbon-based ORR catalysts changes the charge density of the sp^2^-hybridized carbon structure, thus improving the ORR activity^13, 14^. Most NC materials also show a four-electron ORR pathway and have high long-term stability in base electrolyte compared with Pt-based catalysts^13, 15–23^. To prepare NC, several methods have been developed and investigated, including chemical vapour deposition (CVD) using N-containing precursors^13, 15, 16^, heat treatment of carbon under nitrogen-containing reactive gases^17–19^, and the pyrolysis or hydrothermal treatment of carbon materials with nitrogen rich molecules^20–23^. The CVD method allows the *in situ* synthesis of NC, but the need for sophisticated reaction control and the extremely low product yield limit the applications of this technique. The heat treatment method for preparing NC also requires complex instrumentation and toxic gases such as ammonia. In contrast, the pyrolysis and hydrothermal treatments are desirable because of their low cost and mild reaction conditions^20, 21^. Despite many efforts to prepare metal-free catalysts, the electrocatalytic activity of NC toward ORR does not match that of Pt-based catalysts^24, 25^. Therefore, the development of a new non-metal catalyst with high electrocatalytic activity toward the ORR is still required.

Nanodiamond (ND) is a new carbon material with a spherical nanocarbon structure, large surface area, and excellent mechanical and electrical properties^26–28^. ND has many potential applications because of its outstanding properties. In addition, the graphitization of the ND converts it into an excellent material for electrochemical applications^29–36^. The graphitized ND, which is often referred to as an onion-like carbon (nano-onion, NO)^30–32^, provides abundant active sites for the ORR because of its large surface area. However, only a few studies have investigated the ORR activity of NO^34–36^. In addition, the influence of nitrogen-doping onto the NO surface on the ORR activity is still under investigation. In the cost point of view, NO and nitrogen-doped NOs (NNOs) can be inexpensive alternatives to Pt-based catalysts for ORR by using an artificial diamond as a raw material for the fabrication of ND and optimizing the process conditions for the preparation of NNO from ND.

**Figure 1:**
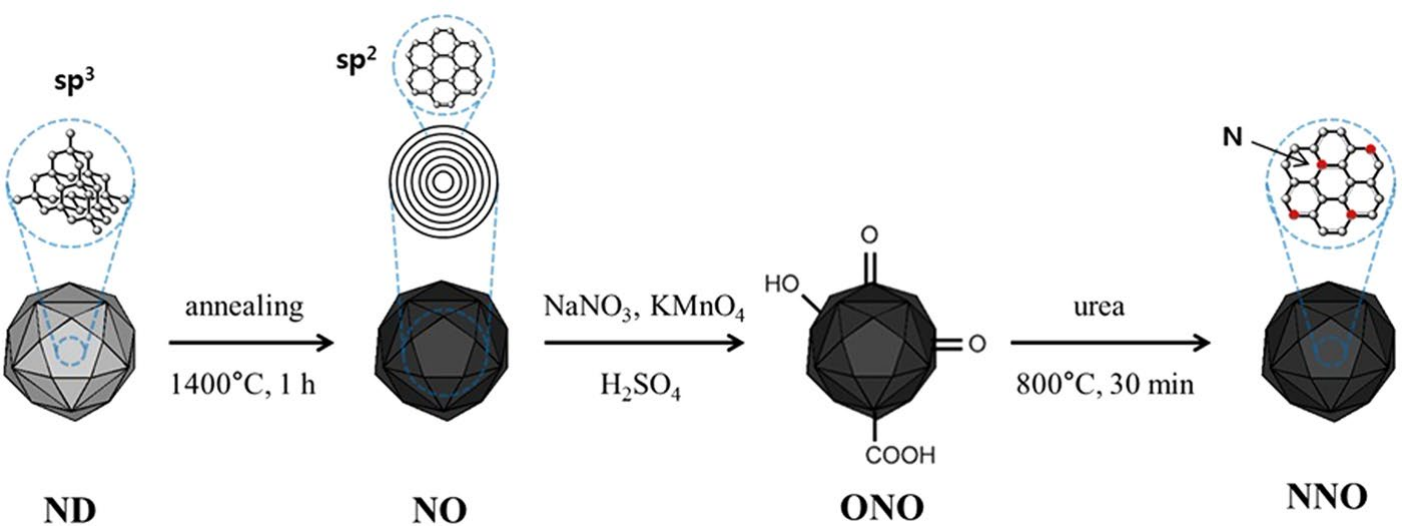
Schematic routes for the fabrication of NO, ONO, and NNO.

In this study, NNOs were prepared, and the changes in the ORR performance of the NNOs were investigated as a function of NNO nitrogen content. As shown in Fig. 1, the NO produced by the annealing of the ND was oxidized by a modified Hummers’ method to obtain oxidized NO (ONO). The oxygen content of the ONO was varied by controlling the reaction time of the modified Hummers’ method. Then, the NNOs containing various amounts of nitrogen were fabricated by pyrolysis with urea. The electrocatalytic activities of the NNOs toward ORR were also explored with a rotating disk electrode (RDE).

## Results and Discussion

### Characterization of ND, NO, ONO, and NNO

**Figure 2:**
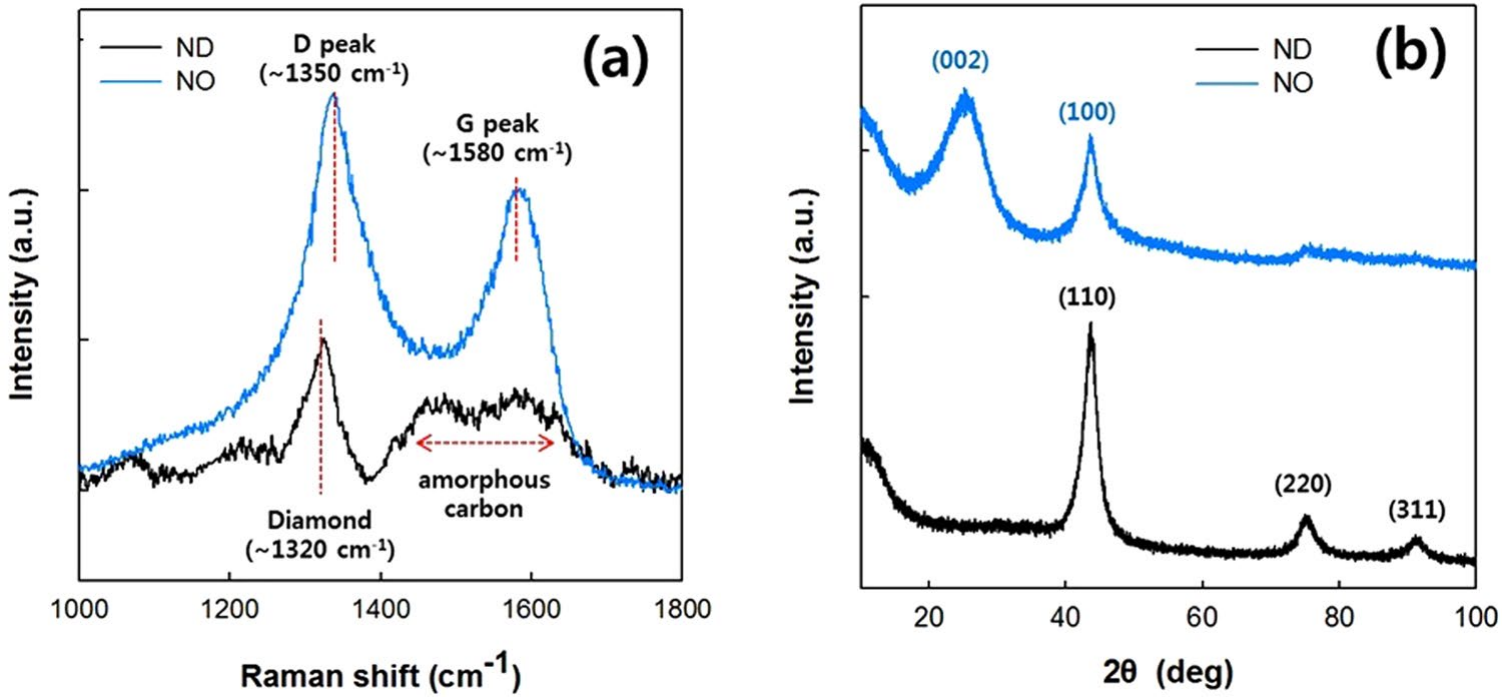
(**a**) Raman spectra and (**b**) XRD patterns of the ND and NO samples.

Formation of the NO was confirmed by Raman spectroscopy and X-ray diffraction (XRD) measurements. As shown in Fig. 2a, the Raman spectrum of the ND exhibited a stretching peak at 1320 cm^−1^, which originates from the diamond structure of the ND^27^. A broad peak at 1400 to 1700 cm^−1^ in the Raman spectrum of the ND is due to the amorphous phase of the NDs, which consists of graphitic fragments and contains surface functional groups^27^. In contrast, the Raman spectrum of the NO showed two stretching peaks at 1350 and 1580 cm^−1^, which correspond to disordered (D peak) and graphitic carbon (G peak), respectively. An increase in the G peak of Raman spectrum of the NO compared with that of the ND indicates that the annealing of the ND at 1400 °C resulted in graphitization. Figure 2b shows the XRD patterns of the ND and NO. The XRD pattern of the ND contains three peaks at 2*θ* = 43.6, 75.3, and 91.4°, corresponding to the (110), (220), and (311) diamond planes, respectively^37, 38^. In contrast, the two peaks at 2*θ* = 25.2° and 43.8° correspond to the (002) and (100) graphite planes in the XRD pattern of the NO. The mobility of the carbon containing groups in the ND surface increases by removing the chemical groups at the ND surface with thermal annealing^39^, then the sp^3^-hybridized carbon structure is rearranged to the sp^2^- hybridized carbon structure.

**Figure 3:**
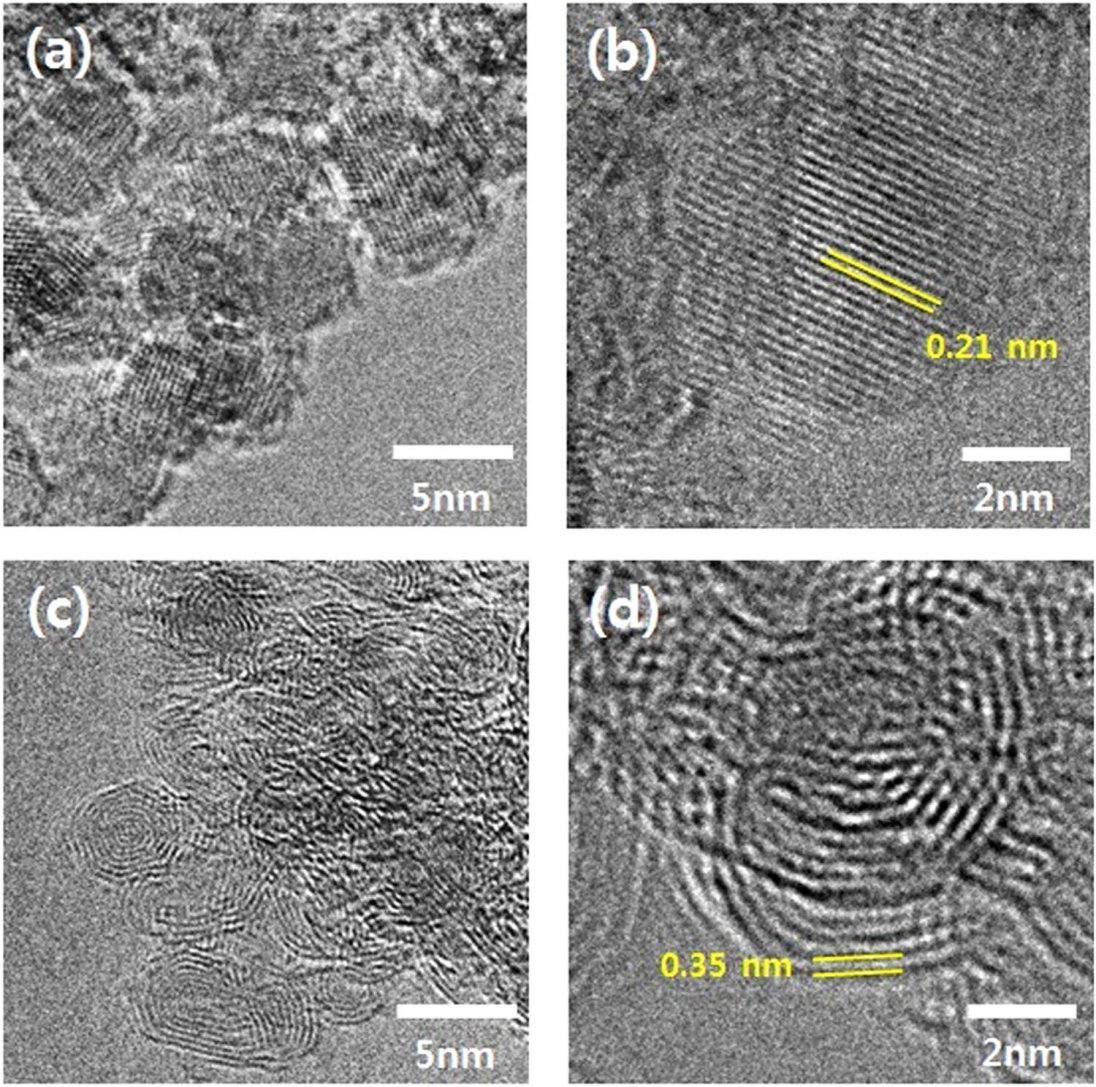
HR-TEM photomicrographs of the (**a**) ND, (**b**) lattice spacing of the ND, (**c**) NO, and (**d**) lattice spacing of the NO.

The ND and NO morphologies were observed by high-resolution transmission electron microscopy (HR-TEM). Figure 3a shows the NDs with particle diameters ranged from 4 to 6 nm. The HR-TEM images of the ND particles at high resolution (Fig. 3b) show the arrangement of crystalline carbon with a lattice spacing of 0.21 nm. In contrast, an onion-like structure with a lattice spacing of 0.35 nm is seen in the HR-TEM images of the NO (Fig. 3c and d), resulting from the graphitization by annealing at 1400 °C. The lattice spacing distances of the ND and NO shown in Fig. 3 are approximately the same with *d*-spacing values calculated from Fig. 2b and correspond to the (110) diamond (2*θ* = 43.6°, *d* = 2.07 Å) and (002) graphite planes (2*θ* = 25.2°, *d* = 3.53 Å), respectively.

**Figure 4:**
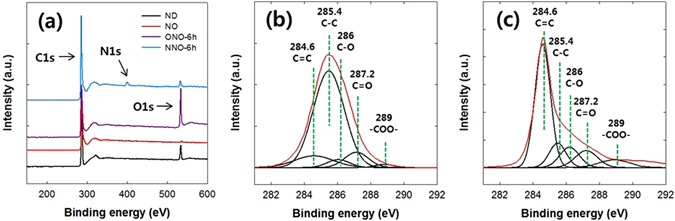
(**a**) XPS wide scan spectra of the ND, NO, ONO-6h, and NNO-6h samples, and (**b**) curve fitting of the C1s peak in the ND and (**c**) that of the C1s peak in the NO.

X-ray photoelectron spectroscopy (XPS) analyses were performed to confirm the formation of the NO, ONO, and NNO. Hereafter, the ONOs are referred to as ONO-1h, ONO-3h, ONO-6h, ONO-12h, and ONO-24h, according to their reaction times in the modified Hummers’ method. The NNOs are also denoted NNO-1h, NNO-3h, NNO-6h, NNO-12h, and NNO-24h in accordance with the ONOs used. Figure 4a shows the XPS wide scan spectra of the ND, NO, ONO-6h, and NNO-6h samples. The chemical compositions of the ND, NO, ONOs, and NNOs are listed in Table S1. The C1s and O1s peaks were observed in the wide scan spectra of the ND, NO, ONO, and NNO. A reduction in the oxygen content of 9.2 to 1.4 mol% was observed when the NDs were transformed to NOs by annealing at 1400 °C for 1 h. The oxygen content in the ONO sample, which was prepared from NO by a modified Hummers’ method, increased with reaction time, up to 6 h, beyond which there was no increase in the oxygen content with increasing reaction time. The N1s peak in the wide scan spectrum of the NNO sample, which was not observed in those of ND, NO, and ONO, indicates the doping of nitrogen on the surfaces of the ONOs. When the reaction conditions for nitrogen doping were fixed at 800 °C and 30 min, the nitrogen content of the NNOs increased with increasing oxygen content in the ONO, as listed in Table S1. The nitrogen content of the NNO was not changed with nitrogen doping conditions. To control the nitrogen content of the NNO, the oxygen content of the ONO was varied by changing the oxidation time. The oxygen containing groups in the ONO are primarily reacted with the urea under the pyrolysis conditions^40^.

Figure 4b and c show the fitting of the C1s peaks of the ND and NO, respectively. The C1s peaks of the ND and NO were deconvoluted into five component peaks at 284.6 (-C = C-), 285.4 (-C-C-), 286 (C-O), 287.2 (C = O), and 289.0 (-COO-) eV. The molar ratios of the chemical groups of the ND and NO calculated from Fig. 4b and c are listed in Table S2. The sp^2^/sp^3^ carbon molar ratio of the NO (67.0/9.4) was much higher than that of the ND (11.2/72.9). This result indicates that the sp^3^ hybridized carbons in the ND are mainly converted to the sp^2^ hybridized carbons in the NO.

**Figure 5:**
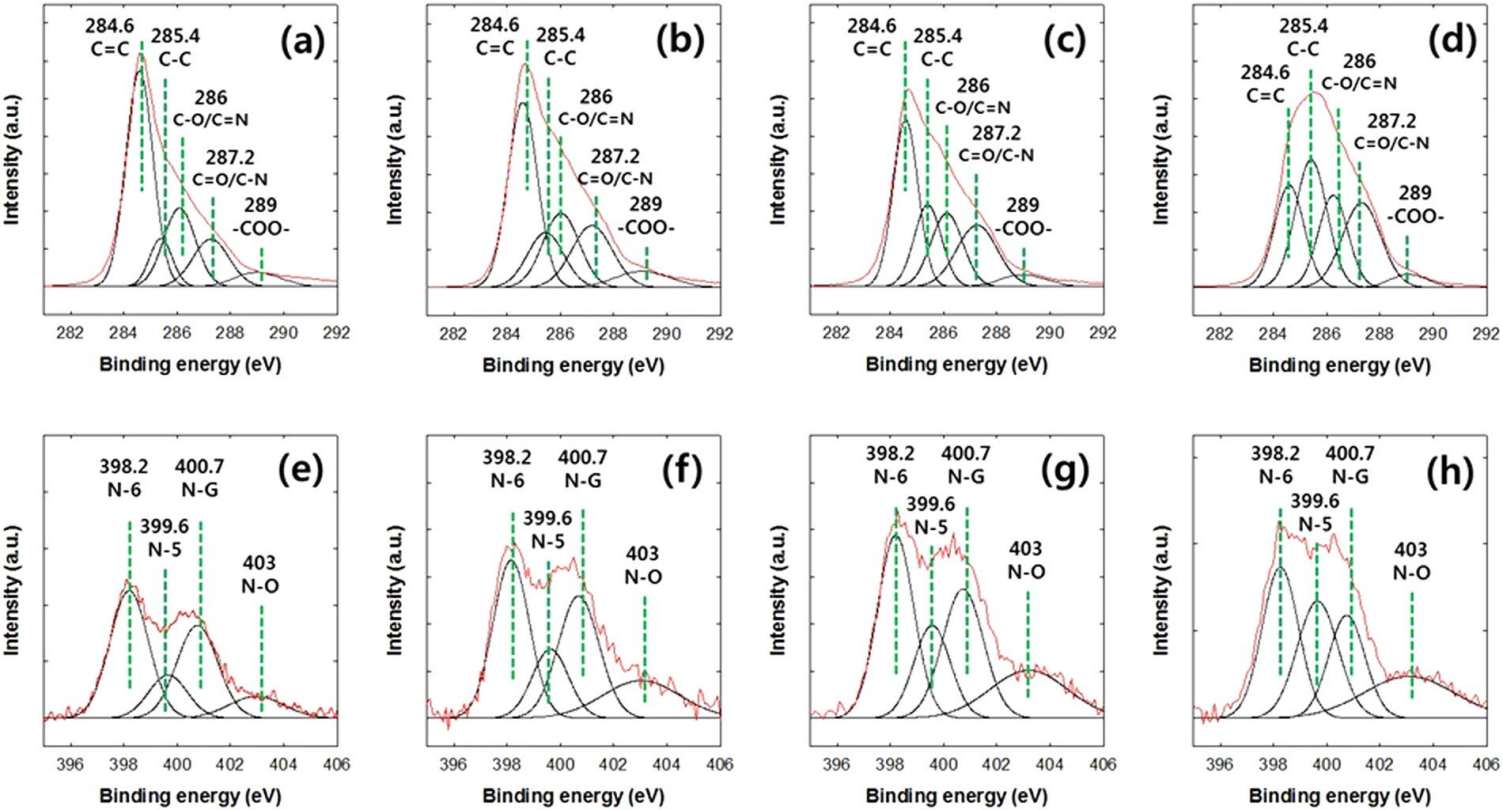
Curve fitting of the (**a**) C1s peak for NNO-1h, (**b**) C1s peak for NNO-3h, (**c**) C1s peak for NNO-6h, (**d**) C1s peak for NNO-24h, (**e**) N1s peak for NNO-1h, (**f**) N1s peak for NNO-3h, (**g**) N1s peak for NNO-6h, and (**h**) N1s peak for NNO-24 h.

The C1s and O1s peaks in the XPS spectra of the ONO-1h, ONO-3h, ONO-6h, and ONO-24h samples were deconvoluted into the component peaks (Figure S1), and the mol% of the chemical groups in the C1s and O1s peaks of ONOs are listed in Table S3. The oxygen content of the ONO was saturated when the reaction time reached 6 h, while the mol% of carbonyl groups increased continuously with increasing reaction time. This increase in carbonyl groups led to a decrease in the mol% of other groups with reaction time. Figure 5 shows the fits of the C1s and N1s peaks of the XPS spectra of the NNO-1h, NNO-3h, NNO-6h, NNO-12h, and NNO-24h samples, and the mol% of the chemical groups in the C1s and N1s peaks of NNO are listed in Table S4. The C1s peaks of the NNOs were deconvoluted into five binding energies at 284.6 (-C = C-), 285.4 (-C-C-), 286 (C-O/C = N), 287.2 (C = O/C-N), and 289.0 eV (-COO-) eV. The N1s peaks of the NNOs were deconvoluted into four binding energies at 398.2 (pyridinic nitrogen, N-6), 399.6 (pyrrolic nitrogen, N-5), 400.7 (graphitic nitrogen, N-G), and 403.0 eV (oxidized nitrogen, N-O). The changes in the mol% of the nitrogen-containing groups in the NNO (See Table S4) influenced the electrochemical performance of the NNO toward ORR, as described in the following section.

Figure S2 shows the FE-SEM photomicrographs of NNO-6h and its EDS elementary mapping for carbon and nitrogen. The doped nitrogen atoms are uniformly distributed on the NNO surface. The surface area and the pore size of the ND, NO, ONO, and NNO were estimated by the Brunauer-Emmett-Teller (BET) method. Figure S3 shows the nitrogen adsorption graphs of the catalysts and the specific surface area, total pore volume, and the average pore diameter calculated from the nitrogen adsorption graphs. When ND was changed to the NO, the specific surface area increased from 343.8 m^2^/g to 435.3 m^2^/g and a subtle increase in the average pore diameter was observed. It is known that the particle density is reduced when the sp^3^-hybridized carbon structure is transformed to the sp^2^-hybridized carbon structure^29^. The specific surface area and the average pore diameter of the ONO were nearly the same with those of NNO, and those of the ONO and the NNO were not changed with oxidation time. These results indicates that the surface morphologies of the ONO and NNO are not changed with the oxidation time and the nitrogen doping.

### Electrochemical performance of the NNOs toward ORR

**Figure 6:**
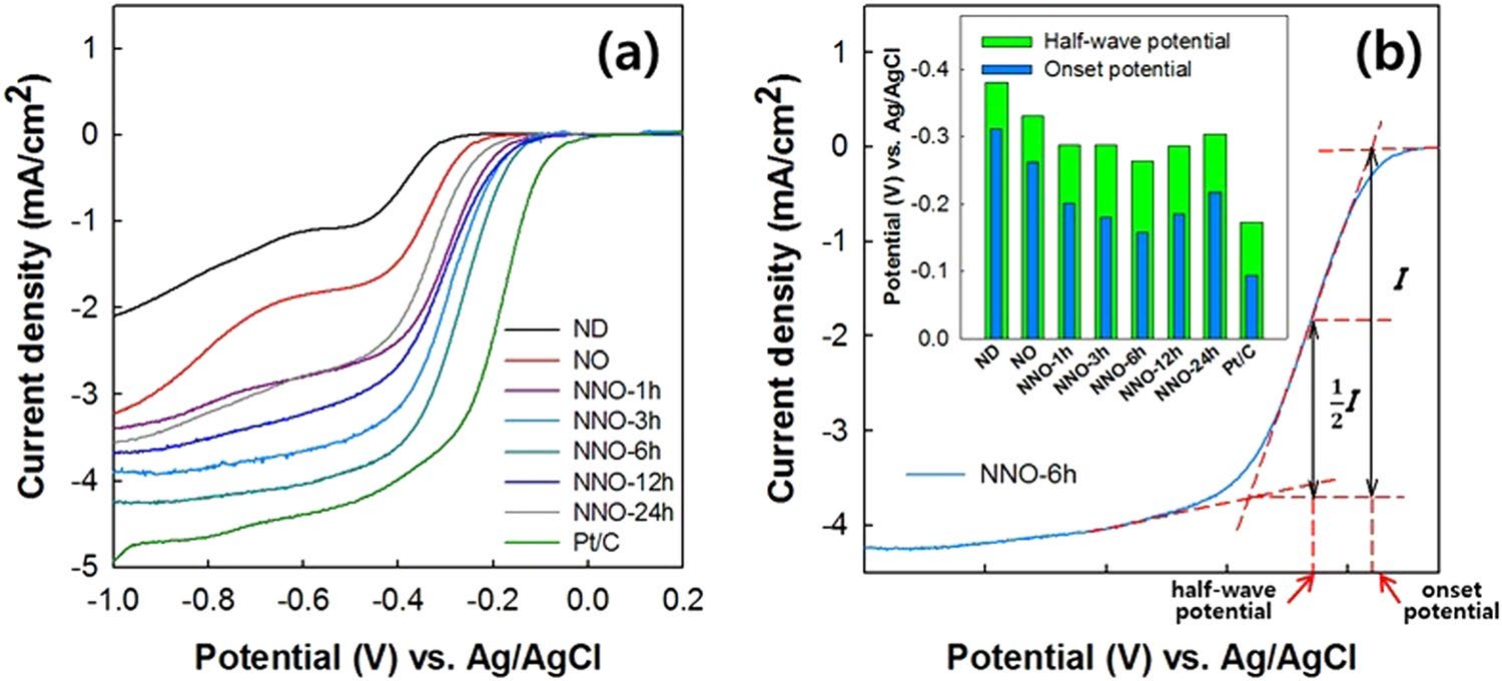
(**a**) LSV curves of the ND, NO, NNO-1h, NNO-3h, NNO-6h, NNO-12h, NNO-24h, and Pt/C samples and (**b**) their onset and half-wave potentials.

Electrochemical measurements of the catalysts toward ORR were performed using the RDE method. Cyclic voltammetry (CV) measurements of the NNO-6h and commercial Pt catalyst supported on carbon black (Pt/C) were performed in an aqueous O_2_-(or N_2_)-saturated 0.1 M KOH solution at a scan rate of 100 mV/s. As shown in Figure S4, the NNO-6h and Pt/C samples both exhibited a cathodic current in O_2_ saturated alkaline media. Linear sweep voltammetry (LSV) measurements were also collected to confirm the ORR activities of the ND, NO, NNOs, and Pt/C. Figure 6 shows the LSV curves of the catalysts in solutions of O_2_-saturated 0.1 M KOH at a scan rate of 10 mV/s at 1600 rpm and their onset and half-wave potentials. The onset, half-wave potentials, and current density of the NO were higher than those of the ND owing to the transformation of the sp^3^-hybridized carbon structure to the sp^2^-hybridized carbon structure. Note that the onset, half-wave potentials, and current density of ONO were nearly the same with those of the NO. This result indicates that the ORR activity of the NO does not changed with the oxidation. The onset, half-wave potentials, and current density of the NO were lower than those of the NNOs. The onset and half-wave potentials of the NNOs were improved in the order of NNO-24h < NNO-1h < NNO-12h < NNO-3h < NNO-6h.

As shown in Fig. 5 and Table S4, the nitrogen in NNO consists of N-6, N-5, N-G, and N-O. Of these, N-6 and N-G re usually regarded as active sites for the ORR^17, 18, 21, 22, 41^. The molar ratio of active sites (N-6 and N-G)/inactive sites (N-5 and N-O) in the NNOs increased in the order of NNO-24h (51.3/47.7) < NNO-12h (58.1/41.9) < NNO-6h (61.8/38.2) < NNO-3h (66.6/33.4) < NNO-1h (77.1/22.9). This result indicates that the molar ratio of the active sites to the inactive sites in the NNO decreased with increasing oxidation time in the modified Hummer’ method. Under fixed nitrogen doping conditions, the nitrogen content in NNO increased with increasing oxidation reaction time (up to 6 h) and then levelled off (see Table S1). Therefore, the absolute number of active sites in the NNO increased in the order of NNO-24h (4.9% nitrogen in NNO-24h × 0.513 = 2.51%) < NNO-1h (2.62%) < NNO-12h (2.85%) < NNO-3h (2.91%) < NNO-6h (3.09%). The electrocatalytic activity of the NNO, including reduction current density and onset and half-wave potentials, also improved with increasing active site content in the NNOs. This result indicates that the electrocatalytic activity of the NNOs depends on the numbers of nitrogen-containing active sites. The functional groups containing oxygen and topological defects in the catalyst also act as active sites^42–45^. As estimated from the BET analyses, the specific surface areas and the average pore diameter of the ONO were nearly the same with those of NNO, and those of the ONO and the NNO were not changed with oxidation time. This result indicates that the ONO and NNO have nearly the same surface morphology regardless of the oxidation time. The similar ORR activities of the NO and ONO also reflect that the effects of the oxygen contained groups in the ONO on the ORR activity are subtle. In summary, the NNO-6h, which contains the greatest number of active sites among the NNOs prepared in this study, exhibited the best performance in the electrocatalytic activity toward ORR.

The number of electrons transfer (*n*) of the NNOs was calculated from the Koutecky-Levich (K-L) equation^46^.

1 $$\frac{1}{J}=\frac{1}{{J}_{L}}+\frac{1}{{J}_{k}}=\frac{1}{0.201nF{C}_{0}{D}_{0}^{2/3}{v}^{-1/6}{w}^{1/2}}+\frac{1}{{J}_{k}}\,$$

In Equation (1), *J*, *J*_L_, and *J*_k_ are the measured current density, diffusion limiting current density, and kinetic limiting current density, respectively. *F* is the Faraday constant (96485 C/mol), *C*_0_ is the bulk concentration of O_2_ (1.2 × 10^−6^ mol/cm^3^), *D*_0_ is the diffusion coefficient of O_2_ in a 0.1 M KOH solution (1.9 × 10^−5^ cm^2^/s), ν is the kinetic viscosity of the solution (0.01 cm^2^/s), and *w* is the rotation speed (rpm).

**Figure 7:**
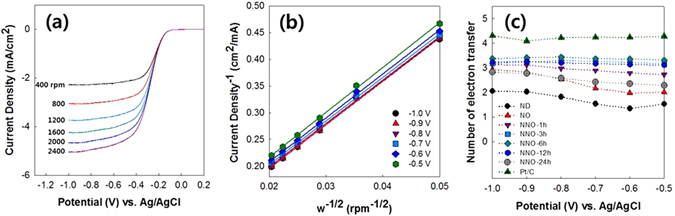
(**a**) LSV curves of NNO-6h examined at various rotation speeds from 400 to 2400 rpm, (**b**) Koutecky-Levich plot obtained from the LSV curves of NNO-6h, and (**c**) the number of electrons transferred for ND, NO, NNO-1h, NNO-3h, NNO-6h, NNO-12h, NNO-24h, and Pt/C.

Figure 7a shows the LSV curves of the NNO-6h catalyst in an O_2_-saturated 0.1 M KOH solution at a scan rate of 10 mV/s under various rotation speeds from 400 to 2400 rpm. The LSV curves of NNO-6h show that their current densities depend on the diffusion of the oxygen with varying rotational speeds. To characterize *n* of the NNOs, we obtained K-L plots from the LSV curves of the NNOs at various rotation speeds. Figure 7b presents the K-L plot of the NNO-6h sample, and its *n* values in the range of −0.5 to −1.0 V were calculated from the slopes of the K-L plot using Equation (1). As shown in Fig. 7c, the NNOs showed higher *n* values than that of NO, and NNO-6h exhibited the highest *n* value among the NNOs studied. Note that the *n* value of the NNOs still does not reach that of the Pt/C catalyst. The unsatisfactory n value of the present NNO might be originated from the lack of the active sites for ORR. An increase of the active sites in the NNO needs to be done in the future work to improve the ORR performance. The number of the active sites might increase by increasing oxygen content in the ONO or changing nitrogen source for the nitrogen doping. The NNO complexes with metal can be promising alternatives in improving ORR activity of the NNO^47–49^.

**Figure 8:**
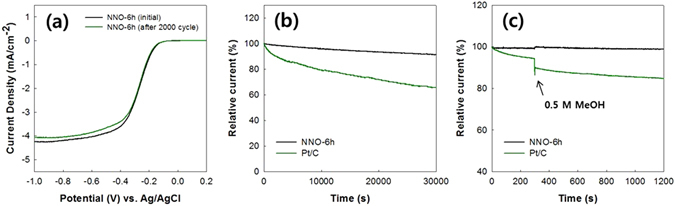
(**a**) Changes in the LSV curves of the NNO-6h after 2000 potential cycles, (**b**) CA responses of the NNO-6h and Pt/C, and (**c**) CA responses of the NNO-6h and Pt/C after the addition of 0.5 M methanol.

The long-term stability of the NNOs and the effect of methanol crossover on the ORR activities of the NNOs were examined. As shown in Fig. 8a, there were no significant changes in the onset and half-wave potentials of the LSV curve of the NNO-6h after applying 2000 continuous potential cycles. The changes in the current density of the NNO-6h and Pt/C were characterized by chronoamperometric (CA) measurements in O_2_ saturated 0.1 M KOH solution at a constant potential of −0.4 V at 1600 rpm. As shown in Fig. 8b, continuous ORR for 30000 s caused a slight loss of relative current in the NNO-6h sample (8.9%); in contrast, a significant loss in the relative current (34.5%) was observed with the Pt/C catalyst. As reported elsewhere^46–48^, the carbon corrosion in the Pt/C during ORR results in the Pt detachment from the carbon matrix. This leads to a decrease of ORR activity in the Pt/C. Figure 8c shows the effect of methanol crossover on the ORR activity of the catalysts. When methanol (0.5 M) was added to the electrolyte at 300 s, the relative current of the Pt/C sample reduced by about 4.9%, while there was little change in the relative current of the NNO-6h sample. Methanol undergoes chemical decomposition on the Pt/C, while it does not undergo chemical decomposition on the NNO^13, 49^. A low reactivity of the NNO with methanol results in better resistance of the NNO toward methanol crossover than Pt/C. These results indicate that the NNO-6h sample has better long-term durability for the ORR and better resistance to methanol crossover than that of Pt/C.

## Conclusions

The NNOs were prepared for use as effective metal-free catalysts for the ORR, and their electrocatalytic activities were investigated by using RDE. The NOs were fabricated by annealing of the NDs at 1400 °C, and their onion-like graphitic structure was confirmed by Raman spectroscopy, XRD, and HR-TEM analyses. Formation of the ONOs and NNOs and analysis of the chemical groups present in these samples were also confirmed by XPS analyses. The oxygen content in ONO increased with increasing reaction time in a modified Hummers’ method for up to 6 h, beyond which there was no increase in the oxygen content with increasing reaction time. When the reaction conditions for nitrogen doping were fixed, the nitrogen content in the NNOs increased with increasing oxygen content in the ONOs. The absolute amount of nitrogen-containing active sites in the NNOs for ORR increased in the order NNO-24h < NNO-1h < NNO-12h < NNO-3h < NNO-6h. The electrocatalytic activity of the NNOs also improved as the content of the active sites in the NNOs increased. As a result, the NNO-6h, which contains the largest number of active sites among the NNOs prepared in this study, exhibited the best performance concerning the electrocatalytic activity toward the ORR. The NNOs exhibited improved long-term stability and resistance to methanol crossover when compared with Pt/C catalysts. In conclusion, although the ORR performance of the NNOs must be improved, their excellent long-term stability and high resistance to methanol crossover indicates that they are effective catalysts for the ORR.

## Methods

### Material preparation

Nanodiamonds (grade: G02, particle size: 4–6 nm), synthesized by controlled dry detonation, were purchased from Plasma Chem. (Berlin, Germany). The NO was fabricated by annealing of the NDs at high temperature. The NDs were placed into a tube furnace in a nitrogen atmosphere and then annealed at 1400 °C for 1 h. The resulting black powder (NO powder) was cooled to room temperature under a nitrogen atmosphere.

The ONOs were fabricated using a modified Hummers’ method. The NO (0.5 g) was dispersed in concentrated sulfuric acid (40 mL) with sonication at 30 °C for 1 h. Next, sodium nitrate (1.0 g) was added to the mixture, followed by the addition of potassium permanganate (4.0 g). The mixture was stirred at room temperature for 1 h, followed by heating at 40 °C. The reaction times at 40 °C were varied for 1, 3, 6, 12, and 24 h to control the oxygen contents of the ONOs. The resulting product was quenched by pouring it slowly into distilled water (100 mL) placed in an ice bath. Then, hydrogen peroxide (10 mL) was added to the solution to terminate the reaction. The ONOs were collected by centrifugation and washed with a hydrochloric acid solution (5%, 2 × 100 mL) and distilled water (5 × 100 mL). The resulting product was dried for 12 h in a vacuum oven at 100 °C.

The NNOs were prepared by the pyrolysis of the ONOs with urea. Urea (2.0 g) was dissolved in distilled water (100 mL). Then, the ONO (0.1 g) was dispersed in the urea solution with sonication for 2 h. The urea solution containing ONO was dried in an air-circulating oven at 100 °C for 2 days until most of the solvent had evaporated. Then, the resulting product was placed in a tube furnace under a nitrogen atmosphere, and pyrolysis was carried out at 800 °C for 30 min to produce the NNO.

### Material characterization

The formation of the NO was confirmed by Raman spectroscopy (LabRAM HR800, Horiba Ltd., Japan) and XRD analysis (D8-Advance, Bruker-AXS, Germany) using filtered Cu-Kα radiation. HR-TEM (JEM-2100F, JEOL, Japan) was employed to investigate the morphologies of the ND and NO. The changes in the chemical composition of the ND, NO, ONOs, and NNOs were explored by XPS (K-Alpha + , Thermo Fisher Scientific, UK) equipped with an Al-Kα X-ray source (1468.6 eV). All peaks in the XPS spectra were calibrated against the carbon (C1s) peak at 284.6 eV, and the widths of the Gaussian peaks were kept constant in each spectrum for curve fitting. FE-SEM/EDS (Sigma, Carl Zeiss, Germany) analyses were carried out to observe the morphology and the distribution of nitrogen atoms doped onto the NNO. Nitrogen adsorption-desorption isotherms of the ND, NO, ONO and NNO were collected at 77 K using an instrument for the BET analyses (3Flex, Micromeritics, USA). The surface area and the pore size of the catalysts were characterized from the nitrogen adsorption-desorption isotherms.

### Electrochemical measurements

The electrochemical characteristics of the NO and NNOs were examined with a three-electrode system. A Pt wire, Ag/AgCl electrode, and glassy carbon electrode (GCE, diameter: 3 mm) were used as the counter, reference, and working electrodes, respectively. The NNO (5 mg) was dispersed in a solution composed of a Nafion solution (5 wt%, 0.5 mL) and distilled water (4.5 mL) with sonication for 1 h. To prepare the NNO deposited GCE, the NNO solution (10 μL) was placed on the GCE and then dried in the air-circulating oven at 80 °C for 1 h. Commercially available Pt/C (20 wt% Pt) catalyst was purchased from Alfa Aesar (Ward Hill, MA, USA) for comparison. The NO and Pt/C placed on each GCE were prepared according to the above procedure.

RDE measurements of the NNO for ORR were performed in a 0.1 M KOH solution using a rotator (RRDE-3A, ALS Co., Japan). The CV, LSV, and CA results were collected using a potentiostat (DY2322, Digi-Ivy, USA). The aqueous 0.1 M KOH solution was saturated with O_2_ by bubbling O_2_ through the solution for 1 h. Oxygen bubbling was continued throughout the reaction to maintain a constant O_2_ concentration in the solution. The nitrogen (N_2_) saturated electrolyte was prepared in the same way for comparison. The CV and LSV measurements were performed at scan rates of 100 and 10 mV/s, respectively, in the potential range of 0.2 to 1.0 V. To obtain the number of electrons transferred from the K-L equation, the LSV measurements were also performed by varying the rotation speed of the working electrode from 400 to 2400 rpm. To investigate the stability of catalysts, 2000 continuous potential cycles were carried out in the potential range of 0.2 to −1.0 V at a scan rate 100 mV/s at 1600 rpm. The CA measurements were also conducted at a constant potential of −0.4 V at 1600 rpm to examine the stability of the catalysts.

## Electronic supplementary material

supplementary information
