# Supplementary material for: Fabrication of nitrogen-doped nano-onions and their electrocatalytic activity toward the oxygen reduction reaction
Source: Sci Rep. 2017 Jun 23;7:4178. doi: 10.1038/s41598-017-04597-6 (PMC5482820; doi:10.1038/s41598-017-04597-6)
Supplement: Supplementary file 1 — supplementary information [file 41598_2017_4597_MOESM1_ESM.doc]

Fabrication of nitrogen doped nano-onion and their electrocatalytic activity toward oxygen reduction reaction

Eun Yeob Choi^1^, C. K. Kim^1*^

^1^School of Chemical Engineering & Materials Science, Chung-Ang University, 221 Huksuk-dong. Dongjak-gu, Seoul, 156-756, Korea

*Corresponding author. Tel: +822 8205324. Fax: +822 8243495, E-mail address: ckkim@cau.ac.kr (C. K. Kim)


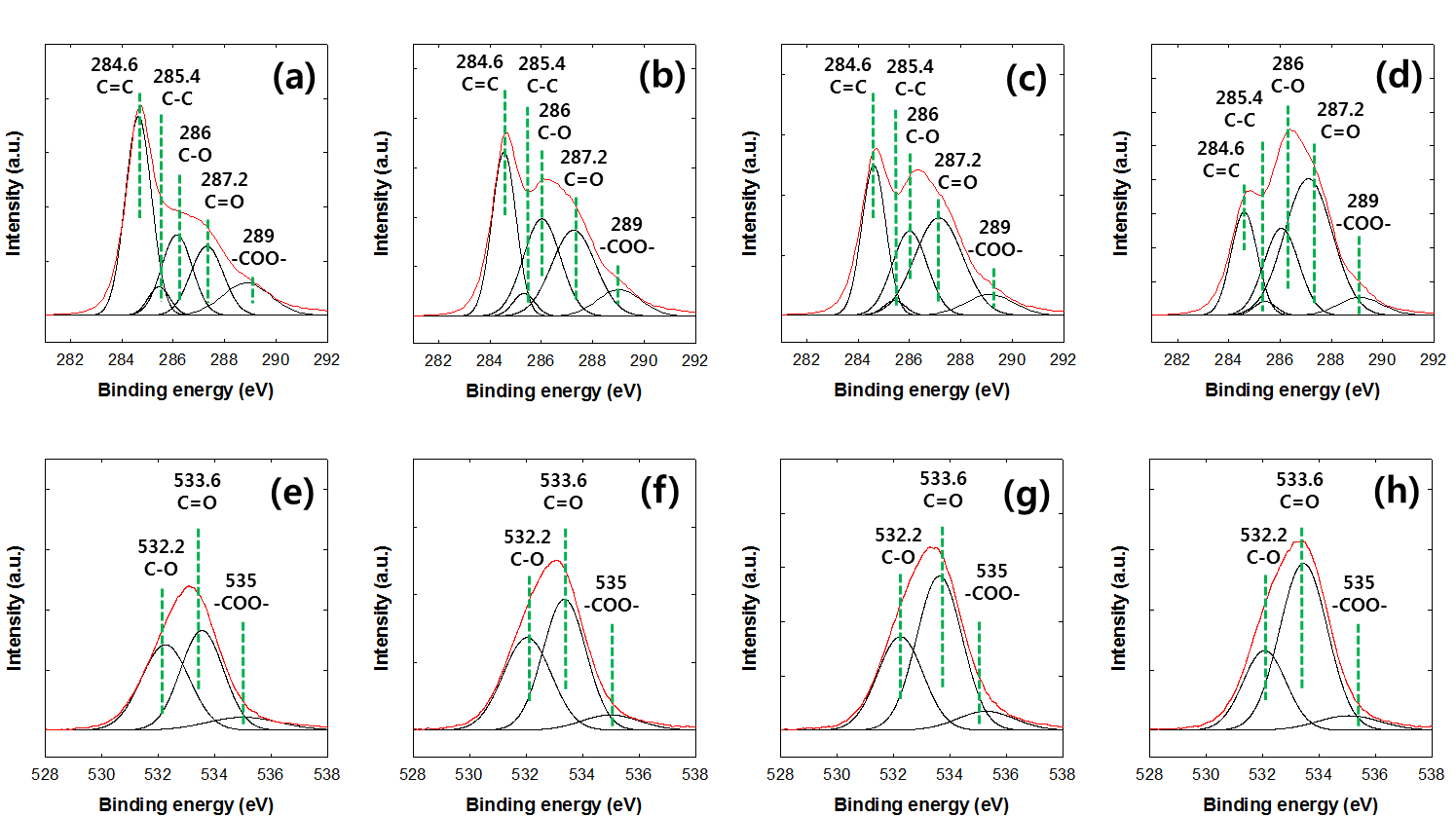


Figure S1. Curve fitting of the (a) C1s peak in the ONO-1h, (b) C1s peak in the ONO-3h, (c) C1s peak in the ONO-6h, (d) C1s peak in the ONO-24h, (e) O1s peak in the ONO-1h, (f) O1s peak in the ONO-3h, (g) O1s peak in the ONO-6h ,and (h) O1s peak in the ONO-24h.


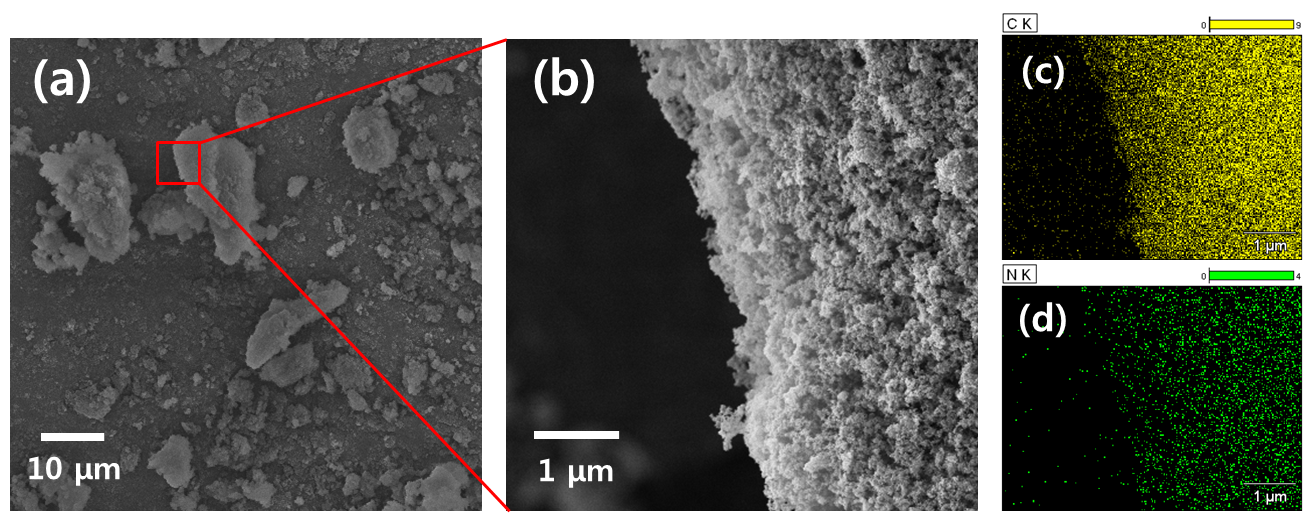


Figure S2. FE-SEM photomicrographs of the (a) NNO-6h and (b) NNO-6h observed at a high resolution, EDS elementary mapping of the NNO-6h for (c) carbon and (d) nitrogen.


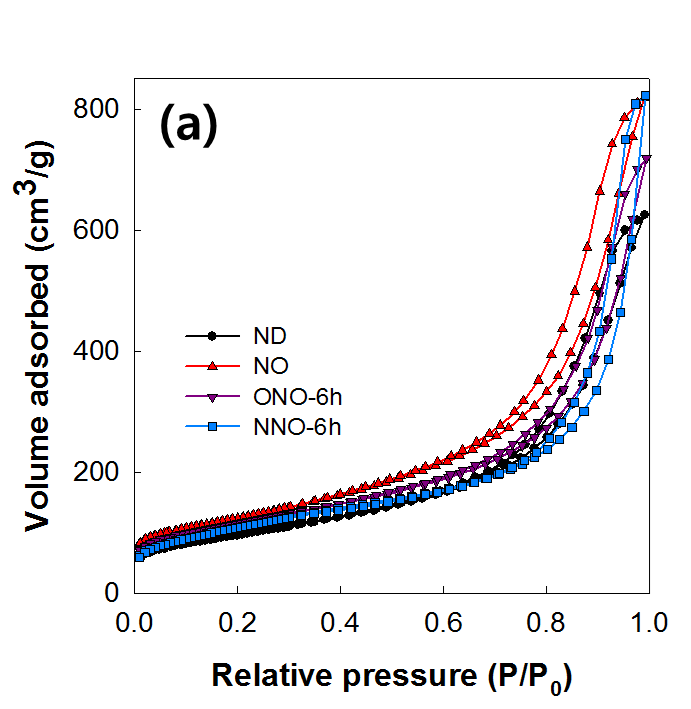


| **(b)** | specific surface area (m^2^/g) | total pore  volume  (cm^3^/g) | average pore diameter  (nm) |
| --- | --- | --- | --- |
| ND | 343.8 | 0.50 | 5.8 |
| NO | 435.3 | 0.68 | 6.2 |
| ONO-6h | 406.4 | 0.58 | 5.9 |
| NNO-6h | 408.9 | 0.62 | 6.1 |

Figure S3. (a) Nitrogen adsorption-desorption isotherms and (b) textural parameters of the ND, NO, ONO-6h and NNO-6h obtained by the BET method.


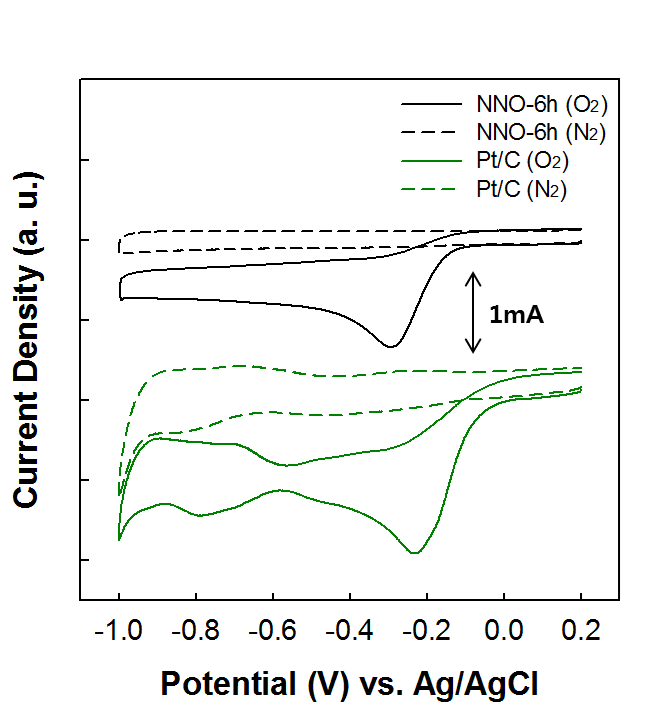


Figure S4. CV curves of the NNO-6h and Pt/C in O_2_ or N_2_ saturated 0.1 M KOH solution at a scan rate of 100 mV/s.

|  | C1s | O1s | N1s |
| --- | --- | --- | --- |
| ND | 90.8 | 9.2 | - |
| NO | 98.6 | 1.4 | - |
| ONO-1h | 86.8 | 13.2 | - |
| ONO-3h | 82.5 | 17.5 | - |
| ONO-6h | 79.7 | 20.3 | - |
| ONO-24h | 80.0 | 20.0 | - |
| NNO-1h | 92.9 | 3.7 | 3.4 |
| NNO-3h | 92.0 | 3.6 | 4.4 |
| NNO-6h | 91.9 | 3.1 | 5.0 |
| NNO-24h | 91.7 | 3.4 | 4.9 |

Table S1. Mole ratio of carbon, oxygen and nitrogen in the ND, NO, ONOs and NNOs

|  | C1s | | | | |
| --- | --- | --- | --- | --- | --- |
|  | C=C (284.6) | C-C (285.4) | C-O (286) | C=O (287.2) | -COO -(289) |
| ND | 11.2 | 72.9 | 4.4 | 10.1 | 1.5 |
| NO | 67.0 | 9.4 | 9.3 | 9.0 | 5.3 |

Table S2. Mole ratio of the chemical groups at the surface of the ND and NO.

|  | C1s | | | | | O1s | | | |
| --- | --- | --- | --- | --- | --- | --- | --- | --- | --- |
|  | C=C (284.6) | C-C (285.4) | C-O (286) | C=O (287.2) | -COO- (289) | | C-O (532.2) | C=O (533.6) | -COO- (535) |
| ONO-1h | 43.0 | 5.1 | 20.3 | 18.7 | 12.9 | | 44.0 | 46.1 | 9.9 |
| ONO-3h | 31.6 | 3.8 | 27.4 | 28.5 | 8.7 | | 40.2 | 51.1 | 8.7 |
| ONO-6h | 28.7 | 2.6 | 23.4 | 37.4 | 7.9 | | 34.9 | 57.3 | 7.8 |
| ONO-24h | 20.5 | 2.3 | 21.6 | 49.7 | 5.9 | | 27.9 | 64.8 | 7.4 |

Table S3. Mole ratio of the chemical groups at the surface of the ONO-1h, ONO-3h, ONO-6h and ONO-24h.

|  | C1s | | | | | N1s | | | | |
| --- | --- | --- | --- | --- | --- | --- | --- | --- | --- | --- |
|  | C=C (284.6) | C-C (285.4) | C-O  /C=N (286) | C=O  /C-N (287.2) | -COO- (289) | | N-6 (398.1) | N-5 (399.6) | N-G (400.7) | N-O (403) |
| NNO-1h | 49.6 | 9.2 | 21.0 | 14.6 | 5.6 | | 43.1 | 11.2 | 34.0 | 11.7 |
| NNO-3h | 40.9 | 14.9 | 20.1 | 18.9 | 5.2 | | 35.6 | 15.4 | 31.0 | 18.0 |
| NNO-6h | 35.4 | 18.9 | 19.8 | 21.3 | 4.6 | | 34.0 | 18.7 | 27.8 | 19.5 |
| NNO-24h | 22.5 | 29.9 | 20.0 | 24.1 | 3.5 | | 30.5 | 26.6 | 20.8 | 22.1 |

Table S4. Mole ratio of the chemical groups at the surface of the NNO-1h, NNO-3h, NNO-6h and NNO-24h.
